# Family Members’ Experiences of Maintaining an Older Relative’s Functional Ability in Long-Term Care

**DOI:** 10.1093/geroni/igaa057.297

**Published:** 2020-12-16

**Authors:** Vilhelmiina Lehto-Niskala, Outi Jolanki, Marja Jylhä

**Affiliations:** Tampere University, Tampere, Finland

## Abstract

Family members have important role in care of older people. In residential long-term settings family members can find themselves in an ambiguous situation: officially, responsibility for provision and quality of care rests with the care provider and staff members, but in practice family members participate in caring. This study explores the role of family members in residential long-term care settings, particularly in supporting their older relatives’ functional ability. Developing and maintaining functional ability lies at the very core of healthy ageing policies and long-term care. The data consist of semi-structured interviews with family members (n=16) from eight long-term care facilities in Finland. Thematic analysis yielded three themes: maintaining personhood, engaging in everyday life and monitoring care. Family members in our study were actively involved in care that supported the functional ability of their older relative. However, family members had also conflicting views about who was responsible for care provision. Some participants willingly accepted their caregiver responsibilities even in residential care, while others described their involvement in care not as a matter of choice but rather as one of necessity in order to ensure good quality care. It is important to see the family members’ viewpoint which, based on the results of this study, emphasizes personhood and continuity of care. If they are willing to participate, family should be able to take part in caregiving together with the care staff and their role should be recognized.

